# Predicting combinative drug pairs towards realistic screening via integrating heterogeneous features

**DOI:** 10.1186/s12859-017-1818-2

**Published:** 2017-10-16

**Authors:** Jian-Yu Shi, Jia-Xin Li, Ke Gao, Peng Lei, Siu-Ming Yiu

**Affiliations:** 1 School of Life Sciences, Northwestern Polytechnical University, Xi’an, 710072 China; 2 School of Computer Science, Northwestern Polytechnical University, Xi’an, China; 3 Department of Chinese Medicine, Shaanxi Provincial People’s Hospital, Xi’an, China; 4 Department of Computer Science, the University of Hong Kong, Hong Kong, China

## Abstract

**Background:**

Drug Combination is one of the effective approaches for treating complex diseases. However, determining combinative drug pairs in clinical trials is still costly. Thus, computational approaches are used to identify potential drug pairs in advance. Existing computational approaches have the following shortcomings: (i) the lack of an effective integration of heterogeneous features leads to a time-consuming training and even results in an over-fitted classifier; and (ii) the narrow consideration of predicting potential drug combinations only among known drugs having known combinations cannot meet the demand of realistic screenings, which pay more attention to potential combinative pairs among newly-coming drugs that have no approved combination with other drugs at all.

**Results:**

In this paper, to tackle the above two problems, we propose a novel drug-driven approach for predicting potential combinative pairs on a large scale. We define four new features based on heterogeneous data and design an efficient fusion scheme to integrate these feature. Moreover importantly, we elaborate appropriate cross-validations towards realistic screening scenarios of drug combinations involving both known drugs and new drugs. In addition, we perform an extra investigation to show how each kind of heterogeneous features is related to combinative drug pairs. The investigation inspires the design of our approach. Experiments on real data demonstrate the effectiveness of our fusion scheme for integrating heterogeneous features and its predicting power in three scenarios of realistic screening. In terms of both AUC and AUPR, the prediction among known drugs achieves 0.954 and 0.821, that between known drugs and new drugs achieves 0.909 and 0.635, and that among new drugs achieves 0.809 and 0.592 respectively.

**Conclusions:**

Our approach provides not only an effective tool to integrate heterogeneous features but also the first tool to predict potential combinative pairs among new drugs.

## Background

The anomaly of the expression level of an individual gene can cause a disease. Specific individual drugs are able to treat the disease by activating or inhibiting the protein regulating the expression of the disease-associated gene. However, the vast number of diseases falls into complex diseases, which cannot be treated by this individual-drug treatment with an expected efficacy [1]. The underlying reason is that complex diseases may involve numerous genes, multiple metabolic pathways as well as diverse environmental factors.

As one of the multiple-target treatments, drug combination has been applied in treating complex diseases (e.g. HIV/AIDS [2] and colorectal cancer [3]) and demonstrated its effectiveness in clinics. However, most experimental approaches of drug combination heavily depend on clinical experience or the test-and-trial strategy. Due to the high cost in both time and money, it is impossible to screen an effective combination of individual drugs among all the possible pairwise combinations on a large scale in wet lab.

Fortunately, the number of available drug combinations is increasing [4]. For example, Drug Combination Database (DCDB) collected 1363 drug combinations (including 330 approved, 1033 investigational, and 237 unsuccessful usages), which involves 904 individual drugs. In addition, a large amount of heterogeneous information (e.g. drug-drug interactions, targets etc.) about individual drugs can be exploited. Thus, it is promising to develop computational approaches to speed up the screening of combinative drug pairs for the treatment of complex diseases [5–9].

Existing computational approaches can be roughly grouped into two types, disease-driven and drug-driven. Disease-driven approaches rely heavily on how well the disease-associated genes or the disease-specific pathways for a disease of interest are known [6, 8, 9]. Diverse assumptions are adopted among them. For examples, (1) two drugs can be combined if their targets are the same or related in terms of the functional pathways of a given disease [6]; (2) the optimum drug combinations can be obtained by maximizing on-target coverage while minimizing off-target effects according to the drug-target network related to the disease-associated genes [8]; and (3) drugs sharing no target or independent signaling mechanisms could be combined, if they have the active functional targets, which are of high-degree and closely connected in the disease-related protein interaction network [9]. For specific diseases, disease-driven approaches are able to predict multiple combinations among drugs. However, it’s hard to integrate other information, such as pharmacology or clinic phenotype, into existing models of current approaches which only use genotype information.

**Fig. 1:**
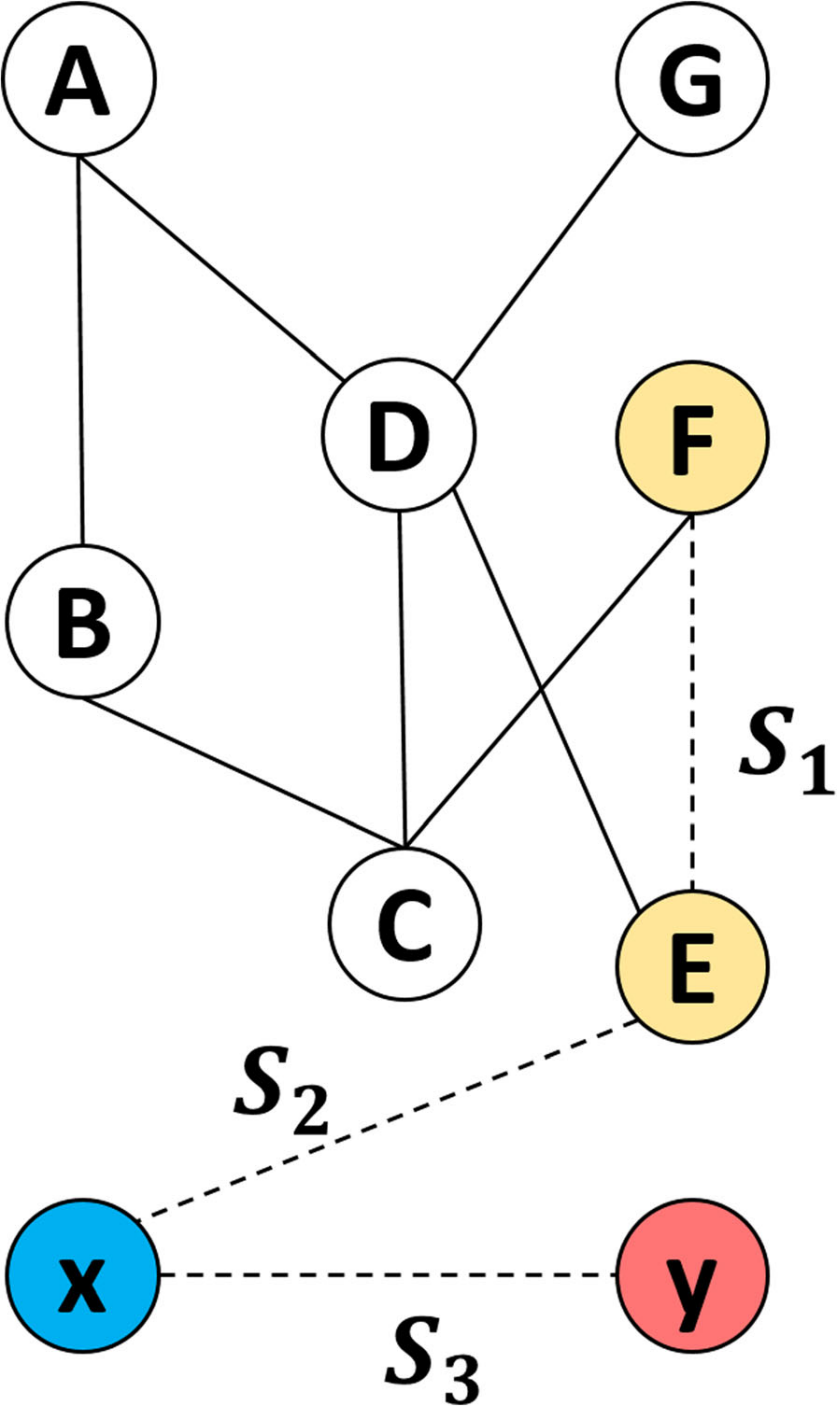
Three scenarios in predicting drug combination. Nodes are drugs, among which known drugs are labeled by ‘A’ ~ ‘E’ and new drugs are labeled by ‘x’ and ‘y’. Edges, represented by solid lines, denote approved combinations between drug pairs. Dotted lines show the three scenarios for prediction corresponding to S1, S2 and S3. The drugs involved in the prediction, are filled with colors

In contrast, focusing on drugs but not diseases, drug-driven approaches are able to predict the candidates of pairwise combinations between individual drugs on a large scale, by holding the underlying assumption that combinative drug pairs are similar to each other and different from ineffective drug pairs. This kind of approaches first represents each drug pair as a feature vector, which characterizes various attributes of the drug pair [5, 7]. Then, varied computational models are built by supervised learning (e.g. frequency-based lazy learning [5] and logistic regression [7]) to predict unknown drug pairs. To achieve better performance, these approaches usually extract drug features from heterogeneous sources, such as ATC codes (drug classification information) and side effects, and concatenate the heterogeneous features into a vector of very high dimension straightforwardly. However, this concatenation leads to a time-consuming training and even results in an over-fitted classifier. More importantly, current drug-driven approaches are narrowly applicable to the drugs having one or more approved combinative drug pairs, but ignore the need of screening potential combinations for newly-coming drugs which have no approved combination at all (see also Fig. 1).

This work develops a novel drug-driven approach. Firstly, we extract four features derived from pharmaceutical drug-drug interactions (DDI), ATC classification codes, targets and side effects. Then, to tackle the abovementioned issues not addressed by former drug-driven approaches, we first design a fusion scheme, which integrates these four features. Then we elaborate appropriate cross validations for three kinds of realistic screening scenarios of drug combinations. Lastly, experiments on real data demonstrate the effectiveness of our fusion scheme for integrating heterogeneous features and its predicting power for not only the drugs having approved combinative partners but also newly-coming drugs that have no known combination. In addition, an extra investigation, inspiring the design of our approach, shows how each kind of heterogeneous feature is related to combinative drug pairs.

## Methods

### Problem formulation

Given a set of *m* known drugs *D* = {*d*_*i*_} , *i* = 1 , 2 ,  …  , *m*, our aim is to predict which drug pairs can be combined together. The prediction of combinative drug pairs can be modeled as a classification problem, by treating all the drug pairs as instances, and known/approved combinative drug pairs as positives and other unknown drug pairs as negatives. Suppose that *d*_*i*_ is represented as an *n*-dimensional feature vector, **f**_*i*_ = [*f*_*i* , 1_, *f*_*i* , 2_,  … , *f*_*i* , *n*_]^*T*^ ∈ **R** ^*n* × 1^, the pair of *d*_*i*_ and *d*_*j*_ is denoted as *c*_*i* , *j*_ = (*d*_*i*_, *d*_*j*_). We believe that two combining drugs have balanced roles in their combination, which is correlated with their synergistic efficacy. Thus, the feature vector of *c*_*i* , *j*_ can be defined as

1 $$ {\mathbf{F}}_{i,j}={\mathbf{f}}_i+{\mathbf{f}}_j, $$

where the addition not only satisfies the symmetry that *c*_*i* , *j*_ = *c*_*j* , *i*_ but also reflects the synergy of these two drugs. After inputting **F**_*i* , *j*_ into a trained classifier, the confidence score of *c*_*i* , *j*_ being a potential drug pair, *Score*_*i* , *j*_, is just assigned with the probability of being a positive instance (see also “Classifier”).

### Feature extraction from heterogeneous sources

We considered four sources of information related to drugs, including pharmacology, anatomy, genotype, and clinical phenotype, which were characterized by drug-drug interactions (DDI), ATC codes, drug-target interactions (DTI) and side effects (SE) respectively.

#### Drug-drug interaction network

Since drug combinations are also called pharmacodynamical or pharmacokinetic DDIs in some contexts. To distinguish drug combinations from pharmaceutical DDIs, DDI in this work only refers to pharmaceutical DDIs, which are usually caused by physical or chemical incompatibility among the co-prescribed drugs.

DDI should be avoided or at least under control if we want to combine the drugs to form a combinative pair. Thus, we extracted this feature based on the interaction matrix between drugs as follows.

Define the adjacent matrix **T** ^*DDI*^ of drug-drug interaction network among *m* drugs, of which *t*_*i* , *j*_ = 1 if *d*_*i*_ interacts with *d*_*j*_, or *t*_*i* , *j*_ = 0 if not. This interaction matrix also represents a drug-drug interaction graph, in which nodes are drugs and edges are their interactions. This graph can be characterized by singular value decomposition (SVD) as follows.

2 $$ {\mathbf{T}}^{DDI}={\mathbf{U}\boldsymbol{\Sigma } \mathbf{U}}^T. $$

Thus, based on SVD, we obtained the feature matrix

3 $$ {\mathbf{f}}^{DDI}=\mathbf{U}\sqrt{\boldsymbol{\Sigma}}, $$

of which the *i*-th row ${\mathbf{f}}_i^{DDI}$ denotes the DDI-based feature vector of *d*_*i*_.

#### ATC-based similarity matrix

ATC classification system divides drugs into a hierarchical classification, according to the organ or system on which they act and their therapeutic, pharmacological and chemical properties [10]. We observed that individual drugs, if combined, tend to work on the same anatomical part in the body (see also “Analysis on heterogeneous features”). Since the first level of ATC code reflects the anatomic properties of a drug and one drug has one or more ATC codes, we calculated the pairwise anatomy-based drug similarities by Tanimoto coefficient as follows and organized them into a semantic similarity matrix **S** ^*ATC*^,

4 $$ {s}_{i,j}^{ATC}=\frac{\left|{A}_i\cap {A}_j\right|}{\left|{A}_i\cup {A}_j\right|}, $$

where *A*_*i*_ is the set of the first-level ATC codes of *d*_*i*_ and |⋅| denotes the size of set.

For example, two drugs, Ondansetron (DrugBank ID: DB00904) and Dexamethasone (DrugBank ID: DB01234), have two sets of 5-level ATC codes {A.04.A.D.12} and {A.01.A.C.02; C.05.A.A.09; D.07.A.B.19; D.07.C.B.04; D.07.X.B.05; D.10.A.A.03; H.02.A.B.02; R.01.A.D.03; R.01.A.D.53; S.01.B.A.01; S.01.C.A.01; S.01.C.B.01; S.02.B.A.06; S.02.C.A.06; S.03.B.A.01; S.03.C.A.01} respectively. Their first-level ATC codes are extracted as {A} and {A, C, D, H, R, S} respectively. Thus, the similarity of these two drugs is 1/6 according to the above equation.

#### DTI-based feature vectors

Former approaches have shown that two drugs can possibly be combined if they target similar proteins, which could regulate the same or similar disease-associate genes [6]. Denote *p* targets interacting with *m* drugs D as *T* = {*t*_1_, *t*_2_,  … , *t*_*p*_}, and the targets of drug *d*_*i*_ as ${T}_i=\left\{{t}_1^i,{t}_2^i,\dots, {t}_{p_i}^i\right\}$, where ${t}_{p_i}^i\in T$, *T*_*i*_ ⊆ *T* and *p*_*i*_ ≤ *p*. We directly used the target profiles of drugs as their feature vector, ${\mathbf{f}}_i^{DTI}=\left[{f}_{i,1},{f}_{i,2},\dots, {f}_{i,p}\right]$, where *f*_*i* , *p*_ = 1 if drug *d*_*i*_ interacts with target *t*_*p*_ or *f*_*i* , *p*_ = 0 if not.

#### SE-based feature vectors

In clinic, a side effect of a drug is an unintended effect, which could be therapeutic or adverse to the host body [7]. Based on our observation that two drugs could be combined if they have many side effects belonging to the set of beneficial side effect patterns (Analysis on heterogeneous features), we adopted the same way as [7] to extract features for drugs as follows. The occurrence of side effects recorded in SIDER [11] can be used as SE features. Thus, similar to DTI, *d*_*i*_ can be represented as a binary vector ${\mathbf{f}}_i^{SE}=\left[{f}_{i,1},{f}_{i,2},\dots, {f}_{i,{n}_{SE}}\right]$, of which *f*_*i* , *p*_ reflects that *d*_*i*_ shows the *p*-th side effect if *f*_*i* , *p*_ = 1, otherwise *d*_*i*_ doesn’t show it.

### Fusion of heterogeneous features

**Fig. 2:**
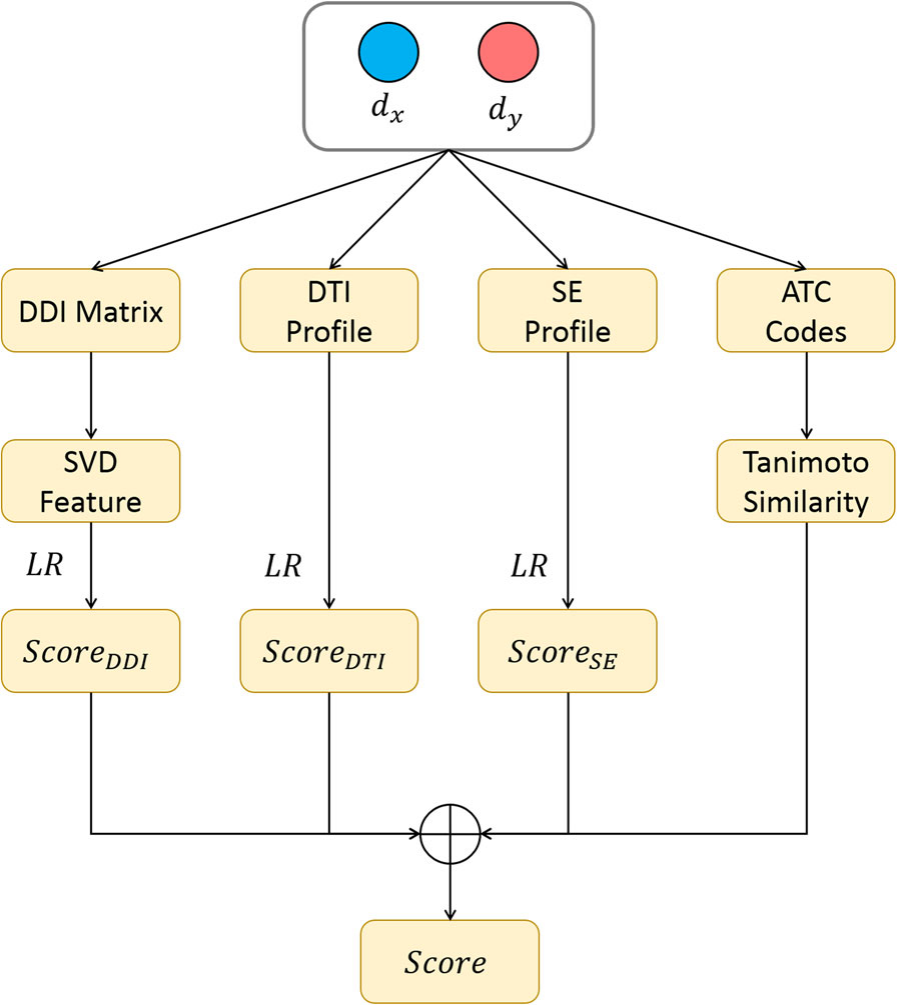
The flowchart of predicting drug combination by integrating heterogeneous sources of drugs. The pair of *d*_*x*_ and *d*_*y*_ is input into three classifier models, which are trained by three kinds of features of drug pairs, including DDI, DTI and SE. The confidence scores of the pair being a potential drug pair are reported by those classifiers and are further integrated with its ATC-based similarity entry. The average of these scores is taken as the final confidence score of indicating how likely the pair can be a combinative drug pair

Drug features not only show the heterogeneity of information source but also have distinct forms in terms of calculation. In details, ${\mathbf{f}}_i^{DDI}$ contains the real-valued features, both ${\mathbf{f}}_i^{DTI}$ and ${\mathbf{f}}_i^{SE}$ are a set of binary, sparse, and high-dimensional feature vectors, and ${s}_{i,j}^{ATC}$ is a form of semantic similarity matrix between drugs. Concatenating all the heterogeneous features into one high-dimensional feature vector would generate computing issues, such as time-consuming training as well as over-parameterized or over-fitted classifier model. Consequently, in order to avoid these issues, we designed a two-step fusion scheme to integrate different drug features and similarity as follows (Fig. 2).

In the first step, the drug pair *c*_*i* , *j*_ of *d*_*i*_ and *d*_*j*_ was input into three classifier models (logistic regression model here), which were trained by three kinds of feature vectors of drug pairs, generated by **f** ^*DDI*^, **f** ^*DTI*^ and **f** ^*SE*^, separately (see also Formula 1). In the second step, its confidence scores of being a potential drug pair, ${Score}_{i,j}^{DDI}$, ${Score}_{i,j}^{DTI}$ and ${Score}_{i,j}^{SE}$, reported by those classifiers. These scores were further integrated with the ATC-based similarity entry ${s}_{i,j}^{ATC}$, which was directly regarded as a confidence score because any similarity function, such as Tanimoto similarity, can be viewed as the decision function of the simplest distanced-based classifiers (e.g. the 1-nearest neighbor classifier) as long as the similarity values fall into [0,1].

According to multiple classifier system, different or same types of classifiers can be integrated together by fusing their output labels or probabilities under various rules. We adopted the Mean rule of fusion to finally average these three scores and one similarity entry to generate the final confidence score of indicating how likely *d*_*i*_ and *d*_*j*_ can be a drug pair.

### Classifier

Logistic regression has been applied in many biological areas, such as combinative drug prediction [7], rare disease variants analysis [12], and disease-gene identification [13]. Predicting potential combinative drug pairs is modeled as a binary classification problem here. Let *C* be the label variable of a drug pair. The label denotes a positive if *C* = 1, otherwise a negative. The logistic model is defined as follows

5 $$ \log \frac{p\left(\mathbf{f}\right)}{1-p\left(\mathbf{f}\right)}={\mathbf{w}}^T\mathbf{f}+b, $$

where **f** is the feature vector, and **w** is the coefficient vector The decision boundary separating positives and negatives is the solution of **w** ^*T*^ **f** + *b* = 0 on the training set of drug pairs.

For a given testing drug pair *dp*_*x*_ and its feature vector **f**_*x*_, its posterior probability of being a positive (a combinative pair) is defined as,

6 $$ p\left(C=1|{\mathbf{f}}_x,\mathbf{w},b\right)=\frac{\exp \left({\mathbf{w}}^T{\mathbf{f}}_x+b\right)}{\exp \left({\mathbf{w}}^T{\mathbf{f}}_x+b\right)+1}=\frac{1}{1+\exp \left(-{\mathbf{w}}^T{\mathbf{f}}_x-b\right)}. $$

Once the classifier outputs the posterior probability, it is directly regarded as the score indicating how likely a drug pair is a combinative pair. Different features (e.g. DDI, DTI and SE) generate different scores, which are further used to measure the performance of prediction in experiments.

### Cross-validation strategies for realistic scenarios

In the screening, one wants to find potential pairwise drug combinations between (S1) between known drugs, (S2) between new drugs and known drugs, and (S3) between new drugs. Here, for short, the drugs having one or more combinations are called known drugs, while the drugs having no combination at all are called new drugs. Three realistic scenarios are illustrated in Fig. 1.

Cross-validation (CV) is the well-established approach to validate the power of generalization of the supervised algorithm in Pattern Recognition. Corresponding to the predicting scenarios (Fig. 1), we designed three strategies (denoted as S1, S2, and S3) of k-fold cross validation (CV) respectively (k = 10 in our experiments). This is important because the appropriate strategies of CV can prevent the computational approaches from reporting the over-optimistic results.

In detail, for the drugs having known combinations, the first CV tries to assess the scenario of predicting new potential combinations among them (S1). For the given drugs having NO known combination at all, the second CV attempts to assess the scenario of predicting new potential combinations between them and those drugs having known combinations (S2). For the given drugs having NO known combination at all, the third CV attempts to assess the scenario of predicting new potential combinations among these given drugs (S3). Thus, though the dataset only consists of drugs that have shown combination with some other drugs, the second and the third CV are still able to indicate how well our predicting approach infers the potential combinations for the new drugs having no combination in practice.

In each round of CV, different scenarios require technically different sets of both training instances and testing instances as follows.

- In S1, we randomly removed 1/*k* drug pairs out of all the given pairs among drugs as the testing instances and selected the remaining pairs as the training instances.
- In S2, we randomly removed 1/*k* drugs out of all the given drugs as the testing drugs and selected the remaining drugs as the training drugs. The pairs among the training drugs were selected as the training instances. Regarding the testing drugs as new drugs, we only selected the pairs between the testing drugs and the training drugs as the testing instances.
- In S3, the training drugs, the testing drugs and the training instances were determined by the same procedure as that in S2. Distinctively, we only selected the pairs among the testing drugs as the testing instances.

In the *k*-fold CV, the above procedures were repeated *k* times and the average of predicting performance in all rounds of CV was taken as the final performance. Two measures were adopted to assess the predicting performance, including the area under the receiver operating characteristic curve (AUC) and the area under the precision-recall curve (AUPR).

## Results and discussion

### Dataset

We adopted the dataset built in [7] as the benchmark, which was collected from Drug Combination Database (DCDB) [4] and FDA orange book [5]. The dataset has 245 drugs containing 239 approved drug pairs (the total number possible pairs is 29,890). These drug pairs are labeled as positives, others are assumed to be negatives for our study.

Four kinds of drug attributes, including ATC codes, target groups, drug-drug interactions, side effects, were utilized to extract the drug features to be used in our approach (see Methods). The first three were collected from DrugBank [14], while the last one originally extracted from SIDER [11] by [7] was directly used.

For those 245 drugs, we firstly extracted their ATC codes. Out of 245 drugs, 150 have one or more ATC codes, of which the codes in the first level were used to calculate drug features. We also applied the ATC predictor, SPACE [15] and are able to obtain predicted ATC codes for 88 drugs having no ATC code. In total, 238 drugs have ATC codes.

Then, we extracted the interactions with targets and other drugs. As a result, 174 drugs out of 245 show 718 interactions with 357 targets, which are given in DrugBank. On the other hand, there are 614 DDIs among the drugs in our dataset, and there exist 8764 DDIs between the drugs in our dataset and 992 extra drugs in DrugBank. After that, we considered 7888 side effects recorded in SIDER for our drugs.

To validate whether the fusion scheme of heterogeneous features, we only picked the drugs having all of ATC, DDI, DTI, and SE features and the drug pairs in which they participate. Finally, our dataset contains 159 drugs as well as 1904 drug pairs, of which 132 known combinative drug pairs are positives and 1772 remaining pairs are negatives.

We adopted logistic regression as the classifier in all experiments and used 10-fold cross validation to assess the predicting performance.

### Feature processing – reducing dimensions

As described in Feature extraction from heterogeneous sources, we generated a set of drug feature vectors. The ATC-based similarity matrix **S** ^*ATC*^ was kept in its original form. We further process the other three kinds of feature vectors as follow. The high dimension of feature vector and the redundancy between features would cause two computational issues: time-consuming training and over-fitted training.

To solve these problems, when calculating **f** ^*DDI*^ by SVD, we discarded the features having the values less than 10^−6^ and obtained 93-dimensional (−d) feature vectors. Since **f** ^*DTI*^ contains 357-d feature vectors, we applied Principal Component Analysis (PCA) to reduce the redundancy between features, so as to reduce its dimension into 131. Similarly, **f** ^*SE*^ contains 7888-d feature vectors, we applied PCA again to obtain 234-d ${\mathbf{f}}_{PCA}^{SE}$. Because the importance of singular values (SVs) and principal components (PCs) is arranged in descending, we just select the first 25 SVs or PCs. Consequently, each feature vector in ${\mathbf{f}}_{PCA}^{DDI}$, ${\mathbf{f}}_{PCA}^{DTI}$ and ${\mathbf{f}}_{PCA}^{SE}$ contains 25 entries finally.

**Fig. 3:**
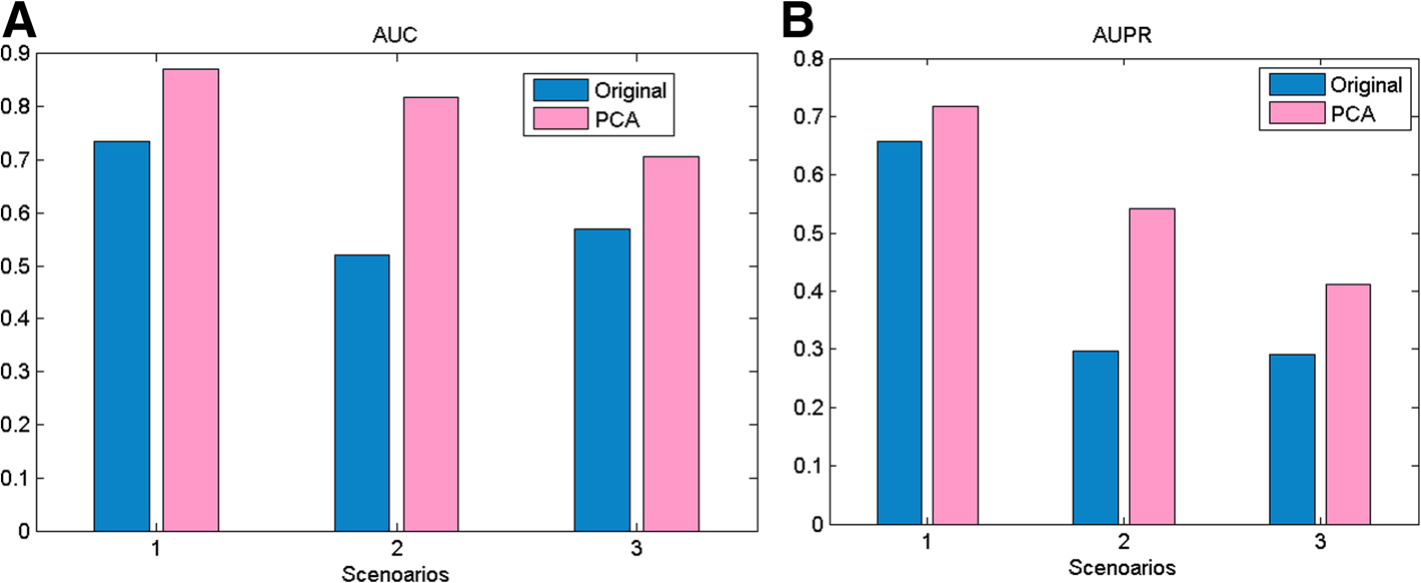
Comparison of the original SE feature vectors and the PCA-processed SE feature vectors. **a** The values of AUC and (**b**) the values of AUPR in three scenarios. Left bars and right bars are the results generated by the original SE feature and the PCA-processed SE feature

The significant advantage of reducing the redundancy between features and the high dimension of feature is the improvement of predicting performance. As an illustration, we compared the results of using 7888-d **f** ^*SE*^ and 234-d ${\mathbf{f}}_{PCA}^{SE}$ in three predicting scenarios respectively (Fig. 3). In terms of AUC and AUPR, the results obtained by ${\mathbf{f}}_{PCA}^{SE}$ is significantly superior to those obtained by **f** ^*SE*^.

### Prediction in different scenarios

We first used four types of features (denoted as DDI, DTI, SE and ATC respectively) to predict drug combination individually, then, upon their predicted scored, we applied the proposed fusion scheme (denoted as *Average* in Table 1) to achieve the better performance. All results are listed in Table 1. In general, SE wins the best feature among four kinds of features, DDI is approximate to ATC, and DTI shows the worst performance. As expected, with the advantage of having low-dimensional features, the fusion scheme under the average rule wins the best performance, and shows a significant improvement, compared to individual features.

**Table 1:** Comparison when using individual features and fusion schemes

	S1		S2		S3	
	AUC	AUPR	AUC	AUPR	AUC	AUPR
DDI	0.816	0.621	0.694	0.343	0.706	0.382
DTI	0.727	0.539	0.737	0.275	0.609	0.210
SE	0.871	0.717	0.818	0.542	0.707	0.411
ATC	0.792	0.393	0.773	0.378	0.708	0.422
Average*	**0.954**	**0.821**	**0.909**	**0.635**	**0.809**	**0.592**
Direct*	**0.955**	**0.830**	**0.910**	**0.644**	**0.809**	**0.592**
Greedy*	**0.955**	**0.837**	**0.916**	**0.669**	**0.834**	**0.605**

The marks * denote three schemes of fusion. The bold entries highlight the results achieved by the fusion schemes

Since the average rule in the fusion step is actually an equal weighting rule, we also investigated whether or not an unequal weighting of those scores can improve the prediction. Two ways to assign weights were adopted. Firstly, the weights of different features were directly assigned according to their values of AUC achieved by performing the prediction individually (denoted as *Direct* in Table 1). Secondly, a greedy search in the scope of [0, 1] with the step of 0.1 were performed to obtain the best weights (denoted as *Greedy* in Table 1). In S1, S2 and S3, the sets of the best weights for DDI, DTI, SE and ATC are {0.4, 0.3, 0.7, 0.4}, {0.3, 0.3, 0.8, 0.6} and {0.6, 0.1, 0.3, 0.5} respectively. Though the unequal weighing is better than the average rule and the greedy search wins the best prediction, they do not outperform the average rule significantly. Thus, the average rule is still an effective approach in practice when integrating various features.

In addition, we investigated the predicting performance by using Support Vector Machines (SVM), in which the kernel function was set with linear function and radial basis function (RBF) respectively. The comparison of using Logistic Regression (LR) and SVM shows LR achieves the approximate performance to SVM-RBF in all the scenarios (Table 2). Besides, LR has an additional advantage of no need to tune parameters.

**Table 2:** Predicting performance with different classifiers

	S1		S2		S3	
	AUC	AUPR	AUC	AUPR	AUC	AUPR
LR	**0.954**	**0.821**	**0.909**	0.635	0.809	0.592
SVM_Linear	0.904	0.639	0.856	0.470	0.720	0.373
SVM_RBF	0.938	**0.821**	0.904	**0.638**	**0.833**	**0.609**

LR is logistic regression, SVM_Linear and SVM_RBF are the SVMs with linear kernel and RBF kernel respectively. The cost parameter is fixed with 100 and the sharp parameter γ of RBF are assigned with 0.02, 0.05 and 0.001 in S1, S2 and S3 respectively when training SVM. The bold entries highlight the best results

Finally, we compared our approach with two existing approaches [16] and [7], of which both model the prediction of drug combination as a classification problem. Considering the concatenation of three kinds of drug features, including chemical interactions between drugs, protein interactions between drugs’ targets and target-enriched pathways, Ref [16] utilizes two techniques of feature selection to choose fewer feature entries and applies Random Forest to predict drug combination. Ref [7] considers two sources of side effects as drug features, including SIDER and OFFSIDES, and directly apply logistic regression on their concatenation to predict drug combination.

However, they do not handle predicting Scenarios S2 and S3. We compared our approach with them in S1 only. Our results are better than [16] (AUC = 0.8803 as stated) and are comparable to the best known results [7] (AUC = 0.92, AUPR = 0.86 as stated in [7]).

The technical difference of our approach to [16] and [7] focuses mainly on two points. Firstly, our fusion scheme provides an efficient framework to integrate heterogeneous features in parallel, so as to enable that the classifiers w.r.t different features are trained simultaneously. Moreover importantly, our approach elaborates appropriate cross-validations towards realistic screening scenarios of drug combinations involving new drugs especially, except for known drugs.

### Analysis on heterogeneous features

In this section, we provided a detailed analysis on how each type of heterogeneous data is related to positive drug pairs. We investigated how well the positive pairs can be separated from the negative pairs when using heterogeneous features (Table 3). In other words, we estimated the separability between positives and negatives. If separability =1, they can be perfect separated, and if separability =0 cannot be separated at all. The detailed investigation is as follows.

**Table 3:** Estimated Separability of positive and negative instances using different features

	DDI	ATC	SE	DTI
Separability	0.6370	0.7218	0.8065	0.5822

Firstly, we built a DDI graph, of which nodes are drugs and edges are their interactions, then applied Flody algorithm [17] to calculate the shortest distance (steps in a graph) between two drugs. The results show that the majority (73.73%) of positive pairs contains the individual drug members apart from 2 steps, whereas only the minority (42.01%) of negative pairs contains the individual drug members apart from 2 steps. Then, we simply estimated the separability between positives and negatives by 0.7373/(0.7373 + 0.4201) =0.6370. In addition, no positive pair has the member drugs are > = 5 steps from each other and very few of positive pairs have the member drugs interacting with each other. This brings the first observation that two drugs do not tend to interact with each other but are usually close to each other in DDI graph if they are combinative. Thus, we used SVD to characterize the DDI graph and extracted the DDI-based feature vectors (see also Drug-drug interaction network).

Secondly, since the ATC-based similarity matrix was calculated directly, we counted the positive pairs, of which its individual drugs share one or more ATC codes, and the negative pairs, of which its individual drugs share no ATC code. The ratio of the former to all the positive pairs (120/132) and the ratio of the latter to all the negative pairs (947/1772) were averaged to estimate the separability (0.7218). This result also brings the second observation that individual drugs in a combinative drug pair tend to act on the same anatomical part in the body.

Thirdly, in terms of the occurring frequencies of individual SE features, we made a statistics on the difference between positives and negatives respectively. It shows that 942 out of 7888 features appear neither in positives nor in negatives, 1344 features occur more frequently in positive, and 5602 features occur more frequently in negative. According to the frequency difference, we may roughly discriminate positive pairs and negative pairs with the separability 0.8065, which is equal to 5602/(5602 + 1344). The statistic shows there are feature patterns to distinguish combinative drug pairs from other drug pairs significantly. Those 1344 features occurring frequently in combinative drug pairs are possibly beneficial to diseases, whereas 5602 features occurring frequently in other drug pairs are possibly adverse to diseases. Thus, the third observation is that two drugs could be combined if they have many side effects belonging to the set of 1344 beneficial side effects.

Lastly, for DTI data, we found that very few drugs pairs (121 out of 1904) share common targets. In details, only 13 out 132 positive drug pairs and 108 out of 1772 negative drug pairs show common targets respectively. Thus, whether or not drugs share common targets, cannot separate positive and negative drug pairs significantly. Considering that all the targets possibly reflect the disease-related pathways, we also made a similar statistics of DTI as that of SE to dig out possible target patterns. The result shows that 53 out of 357 DTI features appear neither in positives nor in negatives, 127 features (positive target patterns) occur more frequently in positives, and 177 features (negative target patterns) occur more frequently in negatives. According to the frequency difference, we may roughly estimate the separability 0.5822, which is equal to 177/(177 + 127). The results reveal the fourth observation that common targets of two drugs are trivial to determine their combination, but these two drugs could be combined if they interact with many positive targets as well as few negative targets.

## Conclusions

Predicting drug combination for complex diseases remains a challenging computational problem. In this paper, we have addressed two issues not solved yet by existing approaches, including an effective integration method for heterogeneous features and the prediction for new drugs (drugs were not used in any drug combination before).

We have proposed four kinds of heterogeneous features (e.g. DDI, ATC, DTI, and SE), in particular, DDI was not considered by existing approaches and we have also presented a new interpretation for the other three remaining features. Based on our four observations, we have provided a clear insight on how these features are related to drug combination. We believe that these observations are beneficial to guide drug combination. Sequentially, we have introduced a fusion scheme to integrate these heterogeneous features with the advantage of low-dimension features used in classifiers.

More importantly, our approach is able to predict potential combinative drug pairs in three realistic screening scenarios involving not only known drugs but also new drugs. Our evaluation results show that the approach is promising. One of the future work would be applying a similar technique to predict more than two drugs that can be combined together.

## Abbreviations

ATC: Anatomical Therapeutic Chemical
AUC: The area under the receiver operating characteristic curve
AUPR: The area under the precision-recall curve
CV: Cross-validation
DCDB: Drug combination database
DDI: Drug-drug interaction
DTI: Drug-target interaction
LR: Logistic regression
PCA: Principal component analysis
RBF: Radial basis function
SE: Side effects
SVM: Support vector machines
